# Impact of selected environmental factors on microbiome of the digestive tract of ruminants

**DOI:** 10.1186/s12917-021-02742-y

**Published:** 2021-01-09

**Authors:** Paulina Cholewińska, Wanda Górniak, Konrad Wojnarowski

**Affiliations:** 1 Institute of Animal Breeding, Wrocław University of Enviromental and Life Sciences, Chełmońskiego 38C, 51-630 Wrocław, Poland; 2 Department of Environment Hygiene and Animal Welfare, Wrocław University of Enviromental and Life Sciences, Chełmońskiego 38C, 51-630 Wrocław, Poland

**Keywords:** Microflora, Digestive tract, Keeping system, Psychological stress, Heat stress, Ruminant productivity

## Abstract

Ruminants are an important part of world animal production. The main factors affecting their production rates are age, diet, physiological condition and welfare. Disorders related to low level of welfare can significantly affect the microbiological composition of the digestive system, which is essential to maintain high production rates. The microbiology of the ruminant gastrointestinal tract may be significantly affected by inappropriate keeping system (especially in juveniles), psychological stress (e.g. transport), or heat stress. This results in an increased risk of metabolic diseases, reduced fertility and systemic diseases. Therefore, the paper focuses on selected disorders i.e., aforementioned inappropriate maintenance system, psychological stress, heat stress and their effects on the microbiome of the digestive system.

## Background

The rearing of ruminants, in particular dairy and meat cattle, is an important element of agricultural production. It is estimated that the number of ruminants worldwide will reach 9.2 billion in 2050. With such rising trend, milk, dairy products and beef are nowadays one of the most desirable products associated with animal breeding. The increase in demand for these products is related to the ongoing steady rise of wealth of societies in developing countries, therefore there is a need to continuously increase production. During intensified breeding, many mistakes can affect the final result, i.e., the animal production index and farm economics. In the case of ruminants, frequent mistakes include inadequate living conditions and unproper diet. These factors have a direct impact on the microorganisms in the digestive tract of ruminants which is reflected in the health status of the animals [1–4].

The digestive system of ruminants is inhabited by many species and types of microorganisms, and their main role is decomposition of nutrients, mainly cellulose and hemicelluloses. Ruminants’ rumen acts as an anaerobic digestion chamber, inhabited in 95% by bacteria, archaea, fungi, protozoa - others are viruses and bacteriophages. The main role of microorganisms in rumen is to convert plant parts of the diet into energy, which would not be possible without their participation. The resulting products are volatile fatty acids (VFA) – mainly propionate and butyrate, which are the main source of energy for the animal and have a direct impact on its physiological parameters, including production rates [5, 6]. Rumen maintained relatively constant pH of 6–7. The main buffers that maintain the pH level in the rumen are potassium bicarbonate and urea. They are found in saliva, which is produced in large amounts by animals, so when swallowed with food, it reaches further parts of the digestive system. The next buffer is ammonia formed during fermentation, which also contributes to the increase in the level of microorganisms in the digestive system [7].

In case of ruminants, not only the composition but also the diversity of microorganisms in the digestive tract play an important role. Ruminants’ digestive tract is estimated to be inhabited by over 5000 species of microorganisms. Their composition depends on factors such as breed, age, external environment and nutrition. The most numerous and most diversely populated is the rumen. Next to the rumen second largest number of microorganisms can be found in the large intestine. They are mainly anaerobic or relatively aerobic organisms, commensals and small quantities of pathogenic microorganisms. Their proper composition and quantity ensures homeostasis of the animal organism, and additionally influences its normal physiological state and level of methane production [8–10]. Bacteria are the most abundant in the digestive system of ruminants. Their quantity and variety are influenced by many factors, such as the composition of the diet, energy requirements and metabolic end products, as some of them may be toxic to certain species. In the case of a diet based on a high content of forage in the rumen, there are more Gram negative bacteria than Gram positive bacteria. On the other hand, in the case of a diet based on high content of grain the situation is reversed [7]. However, in addition to the diet, additional factor that may change the microbiological composition is stress, which may be caused by weaning or changes the environment. The natural protective barrier created by the microbiome, including the digestive system, plays an important role in the development of innate and adaptive immune responses due to the mediation of a number of metabolites derived from microrganisms. Recent studies have shown that there is two-way communication between the microflora of the gastrointestinal tract and the central nervous system. Homeostasis of gastrointestinal microorganisms is threatened by many external factors, the most important of them include heat stress, psychological stress, environment (e.g. maintenance systems) and diet. Therefore, continuous work to understand principal ruminant gastrointestinal microorganisms can have a positive effect on improving the end result in animal production - production rates [11–13].

The purpose of this review was to discuss the impact of selected aspects of environment factors on the microbiological status and production indicators of ruminants.

## Selected environmental factors

The flexibility of ruminants in relation to the food they are consuming has allowed them to inhabit many different habitats covering a wide range of climates. Differences in microbial communities in the digestive system are related to feed conversion and related methanogenesis. Fodders fed to ruminants in various geographic locations differ not only in botanical but also chemical composition, as demonstrated by Henderson et al. [14].

Environmental influence was also demonstrated by Alison et al. [15]. The conducted research shows, on the example of goats, differences in rumen microbiology depending on the environment in which the animals lived. They consisted in isolating a strain of bacteria that are capable of degrading the toxic compound 3 - hydroxy − 4 (1H) - pyridone (3.4 DHP), which is produced in the rumen from mimosine. This non-protein amino acid can be found in the leaves and seeds of the *Leucaena leucocephala* legume shrub which has been often used as a fodder plant for ruminants, in Hawaii. It was not possible to include this plant in the nutrition of Australian goats because the animals could not properly digest mimosine since they did not have in their microbiome composition the bacteria responsible for the decomposition of 3,4 DHP. After introduction of mimosine-digesting bacteria isolated from the rumen of Hawaii goats to Australian goats the use of *Leucaena leucocephala* shrub in nutrition of Australian goats has bacame possible. In this case, the bacterium responsible for the degradation of mimosine was *Synergistes jonesii* (anaerobic Gram negative bacteria).

Welfare is understood as the physiological state of an organism free from physical and mental discomfort, free from hunger and thirst, injury, disease, fear and stress and the ability of animals to express their own behaviour. Unfortunately, the most common cause of the problems in case of farmed animals is desire to obtain high effects at low cost, which is often associated with a reduction and/or disruption of welfare [16–18].

In the case of ruminants, the system of housing affects the welfare of the animals itself, but can also cause the changes in the gastrointestinal tract of the animal of the ruminant. Not only the location of the animals, but also the type of feed, the rearing system, etc., are linked to the system of housing. The study carried out by Fonty et al. [18] on sheep demonstrated that lambs kept in a group experienced a higher increase in cellulolytic bacteria level compared to those kept in single pens, despite being in the same environment and following the same diet. In addition, in the case of dairy cattle kept in the study performed by Gabryszuk et al. [19] it was demonstrated that milk from individuals grazing on species-rich pastures was characterized by higher protein content i.e., α-lactoglobulin, β-lactoglobulin and lactoferrin than milk collected from high yielding cows without an access to pasture. Milk from cows with an access to pasture was also characterized by a different ratio of ω – 3 and ω – 6 fatty acids that was more favorable for humans. In the case of cows with an access to the pasture, the ratio of fatty acids (ω – 3 / ω – 6) was below 1.25, where in case of lack of such access it was 2.5. The presented results were related to the process of protein decomposition in rumen. The organisms responsible for protein decomposition are bacteria and protozoa. Protein-degrading bacteria are mainly anaerobes from *Clostridium* and *Bacilli* phylums i.e. *Clostridium sticklandii, Clostridium coccoides, Eubacterium ruminantum, Lactobacillus fermentum, Proteobacteria (e.g. Ruminobacter amylophilus)* [20, 21].

The protozoa that colonize the rumen (mainly from the *Ciliata* subtype) are responsible, among other things, for providing a full-fledged protein to the host. They absorb amino acids produced by bacteria and digested in further parts of the digestive system. The reduction in the number of protozoa in the rumen has a negative effect on the microbiological structure of the digestive system. They also metabolize excess oxygen entering with food, which is toxic to anaerobic bacteria such as *Eubacterium ruminantum* or *Butyvibrio fibrosolvens* [22, 23]. Therefore, the reduction in their numbers can negatively affect weight gain at the same time lowering the level of protein and fatty acids in milk, which was presented in the works of Jouany [24] and Benchar et al. [25].

It should be kept in mind that housing system that allows animals to graze throughout the entire period of plants vegetation (6 to 11 months) may have a negative impact on the environment, due to methane production by ruminants. Controlling methane production through a properly applied diet is very important issue in terms of environmental protection because it is a gas characterized by high index greenhouse effect. It is 25 times more effective in heat capturing than CO_2_ [11, 12, 26–31]. The main microorganisms responsible for ruminant methane production are archaea. Methane is the final product of both rumen and intestinal fermentation, considered to be a by-product. Archaea colonize the digestive system up to 30 h after birth. They constitute from 3 to 4% of the microorganism population in the digestive system and include species such as *Methanobacterium ruminantum*, *Methanobrevibacter* sp., *Methanosarcina barkeri*. The number and variety of archaea may be related to the diet, environment, health status, and genotype of the animals [7, 32, 33]. It is also speculated that some archaea are related to rumen protozoa. In some studies, such as Jassen and Kris [33], it was suspected that the elimination of protozoa from the rumen was one of the factors changing the composition of the archaea population, both quantitatively and qualitatively.

An appropriate balance of the feed ration in terms of the ratio of roughage to concentrated feed allows to influence the level of archaea in the digestive tract of ruminants., as roughages are more methanogenic than concentrated feeds. The main factor affecting methane production by cattle is the content of propionate producing bacteria, i.e., *Prevotellaceae*, which are more abundant in cows fed with pasture systems [30, 31, 34]. Recent studies have shown that crude fibre is the most methanogenic component in feeds, while crude fat reduces the methanogenesis. A significant factor that also influences the diversity of methanogenic archaea, apart from diet, is the environment in which animals live. The study on sheep showed that both the composition and level of archaea varied from one environment to another [34–43]. However, this is not confirmed in the studies performed by Zhou et al. [38] or Kong et al. [39], however, many studies suggest that future evaluation of the composition of archaea should be based on both species and strain studies, because then there is greater accuracy in determining the occurring changes in their composition.

As mentioned before in the case of ruminants, grazing system allows to ensure a high degree of welfare for the animals, whether in terms of health status, microbiome or behaviour, but may adversely affect the environment. However, not all types of ruminants should be maintained in this system, because the adaptation of animals to the external environment should be taken into account. Adapting animals to the conditions in which they are kept, including investment in nutritional additives, can have a beneficial effect on the microflora of the animals, which in turn can protect them from the effects of digestive system microflora disturbances manifested by metabolic diseases such as acidosis or ketosis. The lack of a suitable diet and maintenance system for animals, especially for high-yielding cows, may also result in disorders of fertility and milk production and the occurrence of, for example, a lameness [11, 16, 39, 42]. However, if the diet is not properly balanced in terms of the level of roughage to concentrated feed in relation to the housing system and taking into account the needs of the animals in a given physiological period, irregularities in the microbiological composition of the digestive system may also occur. Frequent and quick changes in the food dose can disrupt the functioning of the microbiome. Short adaptation time and too rapid changes of feed can result in the depletion of the microbiome of the digestive system, which has an impact on the health status and production rates of animals [2, 6]. Studies on rumen fluid by Hernandez-Sanabria et al.[20], which was collected from animals from various livelihoods, showed that a pasture-based diet increased the number of bacteria from the Bacteroidetes class (mainly the *Ruminococcaceae* family), while a cereal-based diet increased the number of *Prevotellaceae* and *Succinivibrionaceae* (*Proteobacteria*), which is interesting regardless of the ruminant species. Individual bacteria and their metabolism in the digestive system of ruminants may contribute to differences in the level of nutrient absorption from the feed. Changing the diet, i.e. often switching from a low-energy dose to a high-energy feed, can significantly disrupt the functioning of the digestive system microbiome. In such a situation, the value of the feed may be reduced because the microbiome does not fully break down the biomass, which means that less nutrients are absorbed by the host’s digestive system [2, 29].

Research by Mao et al. [44] on goats showed that a diet based on a high content of grains, mainly corn, significantly influenced the structure of rumen bacteria, especially their diversity and composition. Bacteria from the *Firmicutes* phylum predominated with a low number of the Bacteroidetes group in the group of animals fed with a high proportion of grains compared to animals fed with a dose with a higher content of roughage. In this group, the level of protozoa (ciliates) and methanogenic bacteria also increased, and the density of anaerobic fungi decreased. The levels of archaea and toxic and pro-inflammatory compounds have also increased, including endotoxins such as tryptamines, tyramine and histamine. These compounds are abundant in the body and can cause metabolic disorders and even hypertension and neurological disorders [45, 46].

Disorders related to the resulting harmful bacterial metabolites in the rumen can be eliminated by manipulating the microbiome by changing dietary components. Too high level of grains in the diet, exceeding 50% of the food ration, had a negative effect on the microbiological composition and health condition of animals. The unfavorable microbial composition can be improved by increasing the amount of good-quality roughage, thanks to which bacteria from the *Bacteroidetes* phylum will grow [44, 46]. When animals are fed with feed with a high sugar content, e.g. concentrated feed with a high starch content or poor-quality silage, excessive accumulation of lactic acid in the digestive system may occur, which may lead to acidosis. This condition leads to a decrease in the rumen pH from 6.2 to 7.2 to 5, and in the worst cases even to 4. With such a decrease in the rumen pH, as already mentioned, protozoa die, and in the extreme case they are completely absent in the rumen fluid. There is also an increase in the level of liposaccharides in the rumen, as a result of which the population of Gram negative bacteria, mainly from the *Bacteroidetes* group, increases. In the studies of Khafipour et al. [29], when cereal grains were used, the level of liposaccharides in the blood was increased. Increasing their level is associated with the risk of inflammation. However, when administering e.g. alfalfa granules, no liposaccharides were transported into the blood. The above-mentioned authors suggest that the type of high-protein feed used has a significant impact on the occurrence of rumen acidosis. The disturbances in the functioning of the microbiome related to this disease have a significant impact on the general condition of the body, increase the risk of disorders of the reproductive system or even lameness and mastitis [2, 29, 47].

Along with the maintenance system and the diet adapted to it, it is possible to influence not only on the production rates of ruminants, but also the development of juveniles. Young ruminants are exposed to many factors that can influence the microbiological composition of the digestive system. The digestive system microbiome of ruminants develops in the post-prenatal period. During this time, digestive tract is colonized by bacteria (foetal waters, mother’s faeces and milk, external environment). In the first weeks of life, intestinal villi are not fully developed and the rumen does not function, however, microorganisms associated with subsequent rumen fermentation are present, however in a reduced amount. In young ruminants, intense fermentation and significant development of digestive microflora begins with solid food intake. The proper development of the digestive system of ruminants during this period is a physiological challenge for the animal. Its development at this time includes three stages, and each of them determines proper development and future homeostasis of the body [42–49]. The study performed by Tamate et al. [50] showed that feeding of ruminants is highly important during the development of the rumen, the prolonged lack of access to solid feed slows down the development of rumen up to 12 weeks of age, and late development can permanently impair functioning of the rumen. Studies conducted in recent years indicate that a significant proportion of anaerobic bacteria that can be found in adults are already present in the rumen of juveniles between the first and second day of life, although they do not receive solid feed. Tamate et al. [50] carried metagenome sequencing of the rumen fluid derived from 2- and 6-week-old ruminants, and the results of the study showed the presence of glycosidic hydrolases associated with the presence of anaerobic microbiom, even in the absence of a solid diet. Consumption of solid / loose feed allows the development of microflora, as it affects the production of VFA initiating the development of intestinal villi [29, 42].

In the case of the young animals, the microbiological composition of mothers milk, its reproductive system (mainly the vagina) and the oral cavity (saliva - licking after delivery) has a significant impact on the development of microflora. The composition of colostrum and milk at this time also influences the bacterial composition of the digestive system of lambs. During the first weeks of life, the rumen of newborns does not function and the milk passes directly to the abomasum via the intestinal villi. Milk contains a complex and diverse bacterial community, the most abundant are *Bacteroides*, *Staphylococcus*, *Streptococcus*, *Anaerococcus*, *Lactobacillus*, *Porphyromonas*, *Comamonas*, *Fusobacterium* and *Enterococcus* [4, 51]. Urea from milk is converted into ammonia by bacterial ureases because ammonia is necessary for bacteria to produce the amino acids necessary for their proper growth [7].

The results of research carried out so far point out that the balance between the microbiome-host physiology and diet directly affects the successive development and subsequent stability of the resulting microbiome, and thus adequate homeostasis of the animal’s body at a later stages of life [2, 52]. However, so far the mechanisms of microbiome functioning in juvenile ruminants have not been fully understood, and therefore this type of research should still be performed in order to thoroughly understand the effects of microorganisms in the digestive system of juveniles [11, 53].Adaptation of the maintenance system, and concurrently the diet to the type of ruminant (high- yielding, low-yielding, etc.) and the environment in which it resides, can protect the animal against welfare disorders, especially in terms of the occurrence of diseases and associated physical and mental discomfort. The maintenance system affects both young and adult animals, affecting their productivity. The housing system not adapted to the needs of animals is probably one of the factors disturbing the microflora of the digestive system, which may result in the occurrence of diseases, inadequate development of young ruminants [11, 19]. However, further research is needed to show the exact relationship between the maintenance system used and the microflora of the digestive system of ruminants.

## Impact of selected stress factors on composition of the microflora

**Fig. 1:**
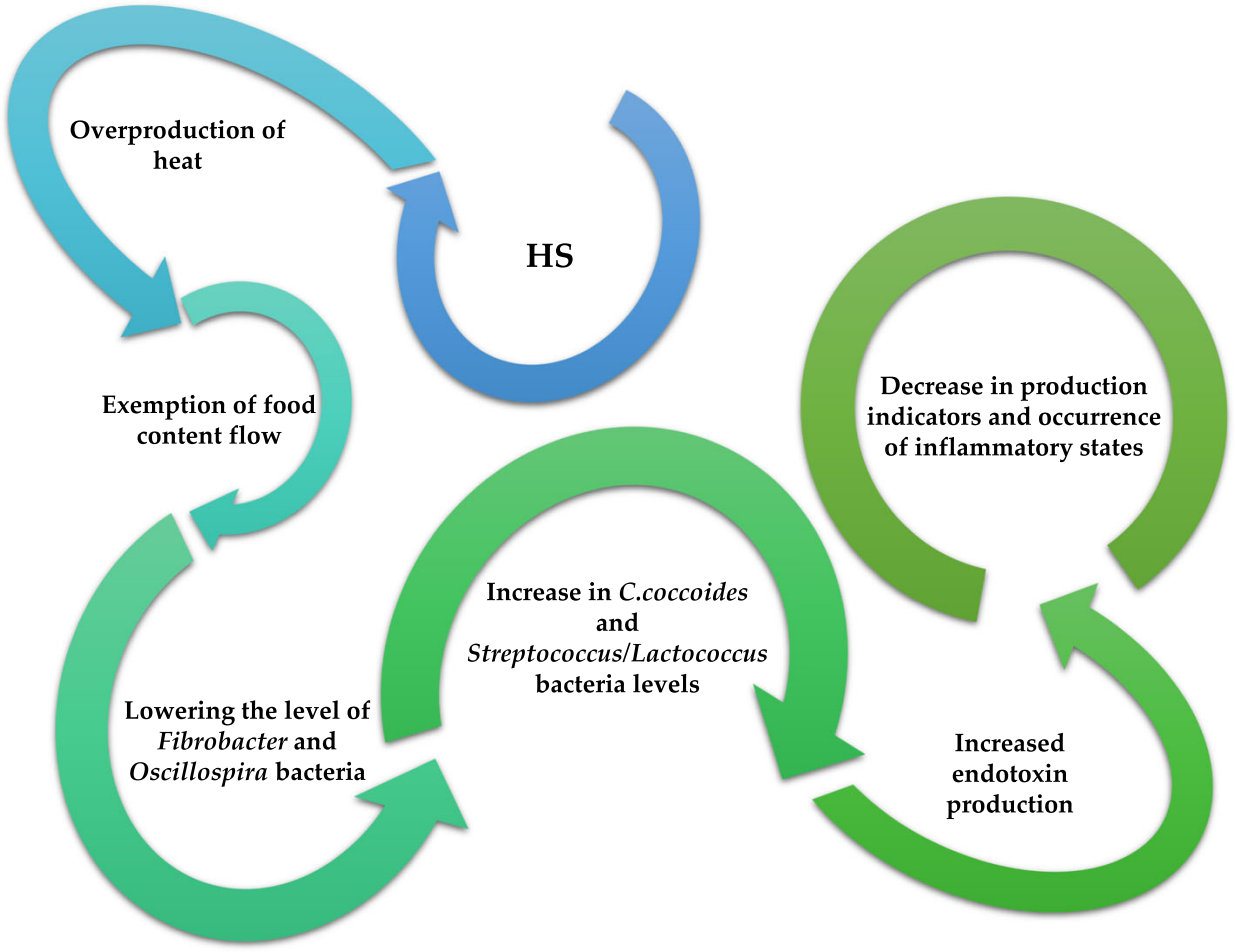
Effects of HS on the health status of ruminants [52, 56, 60]

Modern research shows a significant influence of microorganisms of the ruminants digestive system on their health and productivity. Environmental temperature in combination with humidity also have a significant impact on the microorganisms of the ruminant digestive system. High ambient temperature and humidity may cause heat stress (HS) (Fig. 1). Heat stress consists of factors such as ambient temperature, relative humidity, solar radiation and air movement. The main symptoms of heat stress are increased body temperature and breathing rate, reduced feed intake, increased water intake. In addition, when an animal is subjected to heat stress, significant decrease in animal performance can be observed. Considering the warming of the climate, the problem of heat stress affects increasing number of animals, including ruminants. HS is often considered by the breeders as one of the main obstacles to efficient livestock production [28, 54–58]. Heat stress mainly affects dairy cattle because they are additionally burdened with milk production and often poorly adapted to heat stress conditions. The consequences of heat stress are significant fertility problems. Cows exposed to high ambient temperatures demonstrate a significant increase in overall fatigue, an increase in the number of inseminations per cow, an increased incidence of early embryonic mortality and an extension of the post-natal and inter-calving period [28, 55, 58, 59]. During this time, metabolism is subject to an increase, which significantly affects microorganisms in the digestive system [54]. In addition, animals do not mobilise adipose tissue during exposure to heat stress, although they have a negative energy balance and catabolic status [55]. So far, many studies have been carried out to characterise and mitigate the effects of heat stress, however, there is still little information on changes in the gastrointestinal microbiome, but it has been found that it impairs the integrity of the digestive system [55, 56].

Ruminants’ response to heat stress involves reduced uptake of dry matter in feed in order to reduce the metabolic production of heat, and maintain a constant temperature. In addition, preference is given to the consumption of more concentrated feed than rohughages. Such a change in diet may cause acidosis, as an increased uptake of concentrated feed is disturbed by the fermentation process in the rumen. In this situation, the level of *Fibrobacter* and *Oscillospira* bacteria decreases, while the level of the *Clostridium coccoides* and *Streptococcus*/*Lactococcus* genera increases, the production of short chain fatty acids (SCFA) and the level of acetate decreases, while the level of propionate and lactate increases. Lactate is often produced by bacteria, i.e., *Streptococcus bovis*, and in comparison to acetate, propionate or butyrate, it is hardly absorbed by the rumen epithelium, which may cause a decrease in the rumen pH (about: 6.8–6.5). Depending on the humidity - the higher the humidity, the more significant the pH drop will be. In addition, in the event of heat stress, there is an increase in water consumption in relation to the dry matter of the feed, which results in a significant slowdown in the flow of food content causing an increase in the acidity of the rumen fluid due to the extended rumen retention [53, 56, 61]. In the study by Tajima et al. [61] demonstrated that HS significantly influenced the microbiome diversity of the digestive system of ruminants and production rates. Body weight at 33 °C and over 80% humidity were more than half lower than at 20 °C. There was also a reduction in the consumption of dry matter, in the case of 33 ° C and humidity at the level of 80%, even by half. As for microbiological changes, they were most visible in younger animals at 60% humidity and 28 ° C. In this case, there was an increase in the level of the *Bacteroidetes, Spirochetes* phylum and a decrease in the level of *Firmicutes*.

The study performed by Contreras-Jodar et al. [57] demonstrated a significant influence of heat stress on microbiological homeostasis of the digestive system as well as milk yield and composition in goats; there were some changes in the thermo – physiological and milking characteristics of goats. An increase in the excretion of toxic compounds by digestive microorganisms and a decrease in the bioavailability of aromatic amino acids was observed. Heat stress also disturbed the hormonal balance of animals, mainly the synthesis of thyroid hormones and the action of neurotransmitters, i.e., levodopa, serotonin. Hormonal imbalance and disruption of neurotransmitter function results in a decrease in milk production and a change in its composition. Another study by Chen et al. [62] shows the important effect of microorganisms on the cerebral-intestinal axis. The results show an increase in cortisol and cytokines in milk plasma in HS dairy cows. However, plasma levels and oxytocin as well as triiodothyronine and thyroxine decreased. Dairy cows subjected to HS decreased the diversity of the microbial population, which resulted in greater expansion of pathogenic microorganisms, the adaptation pathway to the environment slowed down and the immune response pathway and metabolic pathway were disturbed. Exposure to the HS environment modulates the physiological characteristics of the animal, which may result in changes in the composition and amount of microorganisms in the digestive system.

In addition to temperature and humidity and the adverse conditions associated with them, another type of stress can be another environmental factor affecting the microflora of the digestive system. One of the stress factors in farm animals is transport. During this time, the animals are exposed to very strong stress related to the change of environment and means of transport, as well as to thermal stress and fatigue [63]. In the study performed by Deng et al. [64] on the beef cattle, it was demonstrated that changes in the environment related to the transport of young animals have a significant impact on the relationship between levels of different groups of bacteria. During 6 h after the transport, the number of *Fibrobacter succinogenes*, *Ruminoccocus flavefaciens*, *Ruminococcus amylophylus* and *Prevotella albensis* as well as rumen acid production was subject to an incrae, and concurrently a decrease in *Prevotella britain*, *P. ruminicola*, *Anaerovibrio lipolyitica*, *Succinivibrio dextrinosolvens* number, rumen pH and rumen butyric acid production can be observed. It was only after 15 days that the physiological processes and the level of bacteria in the rumen were stabilized. Such a long-lasting decrease in the pH level may cause a decrease in the level of protozoa, which can reduce the level of feed protein utilization. Protozoa are the main source of full-value protein for ruminants, so their reduced amount may adversely affect growth during the adaptation period of ruminant microflora after a change in the environment [25, 26]. Microbiome of the digestive system plays an important role in the development of innate and adaptive immune responses via a number of metabolites derived from them [52, 59, 65]. The pathways of communication between the microbiota and the brain include the vagus nerve, intestinal hormone signaling, the immune system, tryptophan metabolism, and microbial metabolites such as short-chain fatty acids. Animal studies have shown that early separation from the mother, as well as chronic stress, lead to intestinal dysbiosis, unsealing of the intestinal barrier, and disturbances in the endogenous synthesis of tryptophan, dopamine and serotonin. The effect of which may be an increased concentration of cortisol and depressive or anxiety behaviors [66, 67].

The disorder under the influence of psychological stressors provokes a neuroendocrine response, which may have a direct or indirect effect on the microflora of the digestive system at the same time, in which case the inflammatory markers may increase in the absence of overt infection. The digestive microbiome plays a key role in regulating the pathway between the brain and the gut. During this time, the level of bacteria from the *Lactobacillaceae* family and other conditional aerobes may fall, which may result in an increased risk of diseases caused by pathogenic microorganisms [67, 68]. Prolonged psychological stress causes a significant reduction in mucin production and an increase in the presence of acid mucopolysaccharides on the mucosal surface, which facilitates colonization of the intestines by pathogenic organisms e.g. *Escherichia coli* O157:H7 or *Salmonella enterica*. The studies conducted so far indicate that long-term stress (at least 4 hours) determines the production of adhesins by pathogens. It is associated with a long-term increase in the level of catecholamines - stress hormones and an increase in the level of toxins (e.g. shiga). If an animal is subjected to prolonged acute stress, bacterial translocation may also occur, which also causes catecholamine levels. Most often, translocation occurs to the lymphatic system, however, in the worst case, it may occur to the circulatory system, which in turn may result in e.g. sepsis [69].

The role of the microbiome in gut-brain interactions was first discovered in the GF mouse study. These mice were overactive to stress in the hypothalamic-pituitary-adrenal (HPA) pathway compared to specific pathogen-free control mice. The reconstruction of *Bifidobacterium infantis* reversed the HPA stress response in GF mice [70–74]. Working with germ-free (GF) mice (i.e., born surgically and raised under sterile conditions) shows a link between the microbiota and anxiety-like behaviors [66]. In the studies by Sudo et al. [69] where the level of stress response in germ-free (GF), specific pathogen free (SPF) and gnotobiotic mice was compared, it was shown that GF mice showed increased levels of ACTH and corticosterone in response to stress compared to SPF mice. In addition, microbiological modification by administering the *Bifidobacterium infantis* strain to GF mice resulted in a weakening of the stress response. However, it was only after the microbial composition of the digestive system was similar to that of the mice that SPF significantly reduced the stress response of the GF mice. Similar effects in their studies were received by Liu et al.[75] and Desbonnet et al. [76]. Recently, it has been proven that there is communication between the microflora of the digestive system and the central nervous system, which is why it is believed that physiological and mental stress disrupts not only the immune system, but also hormonal homeostasis and digestive microflora. Balanced intestinal microflora is important not only for maintaining intestinal homeostasis, but also for regulating the functioning of the immune system and has a direct effect on the entero-cerebral axis [59, 68, 74–77].

It has also been proven that the microbiome of the digestive system can influence, inter alia, the regulation of the level of intestinal peptides, which can discharge into the vagus nerve pathway and affect the intestinal metabolism. The digestive tract is densely innervated by a network of 200–600 neurons that make up the enteric-nervous system. Certain vagal neurons produce receptors for intestinal peptides such as CCK, ghrelin, leptin, PYY, GLP − 1, 5 - HT, which the intestinal microflora can regulate to influence nutrient metabolism. In addition, SCFA, TMAO and IgA bile acids are known to regulate metabolism through the microbiome - gut - liver axis [70]. The liver - microbiome axis is based on communication through the portal vein, biliary tract and systemic circulation. The gut-brain axis, on the other hand, operates through five two-way communication pathways: the neuroanatomical pathway, the neuroendocrine axis (HPA) pathway, the immune system, the microflora metabolism pathway, and the intestinal mucosa barrier along with the blood-brain barrier (BBB). Communication between the microbiome and the brain is related to the regulation of the metabolic state and health of the host, through the production of short-chain fatty acids by the microbiome. They influence the regulation of glucose and lipid metabolism as well as the immune system responses, including inflammatory ones. In the case of internal neural networks in the colon, unique transcription profiles have been demonstrated, which are controlled by the combined effects of genetic programs in the host and microbial colonization. Additionally, it has been shown that the depletion of the digestive system microbiome reduces the excitability of intestinal neurons, slows down intestinal peristalsis and extends the time of intestinal transit. An experiment carried out on mice showed that the digestive system is equipped with molecular mechanisms that monitor the state of the intestinal lumen, thanks to which the activity and mobility of neurons is adjusted accordingly. However, these mechanisms are not fully known [70, 73, 74, 77].

Ensuring proper welfare in terms of environmental conditions and the ability to reduce psychological and physical stress is associated with production efficiency. Changes in the microbiome of the digestive system in terms of stressors can cause a significant decrease in animal productivity, which will be reflected in the economy of the farm, due to a decrease in ruminants productivity. This type of problems most often are observed on the large-scale farms, where due to the large number of animals and maximization of production, the quality of life of the animals is reduced. The more intensive farming is, the more level of welfare decreases. One of the keys to success may be to ensure optimum welfare at maximum production, which will also be visible in the stabilization of digestive tract microflora, and thus improvement of ruminant productivity [59, 64–70, 78–80].

There are also some research evidence from studies performed on single-stomached animals by Aldritt et al. [81] suggesting that during transformation of plant derived compounds, by-products of given transformation can be fixed in tissue of living animal (i.e. bones). Such possibility opens a whole new field for confirmation of such mechanism taking place in ruminants and observation of possible effects or host-microbiome interactions.

## Conclusions

Reducing the occurrence of stressful situations, both physiological and psychological ones, is one of the important aspects of well-being affecting microbiology of the digestive system. A disturbance or reduction of animal welfare may cause irregularities in the functioning of the microbiome and pathway of the digestive system – the brain. The effects of such disorders may be decrease in ruminant productivity as well as metabolic and systemic diseases. Additionally, the animal maintenance system may disturb the aforementioned pathway due to the lack of adaptation to the housing systems applied for certain species or breeds of ruminants. This can cause mental and physical discomfort, which results in a decrease in production indicators and gastrointestinal microbiom disorders. The housing systems chosen by breeders can also affect the development and stabilization of juveniles, which can contribute to an increased risk of metabolic and systemic diseases later in life. Therefore, maintaining optimal welfare is important for animals but also for breeders in economic terms.

## Data Availability

Not applicable.

## Abbreviations

HS: Heat stress
VFA: Volatile fatty acids
HPA: the neuroendocrine axis
GF: germ – free
SPF: specific pathogen free
ACTH: adrenocorticotropic hormone
CCK: cholecystokinin
GLP – 1: glucagon-like peptide-1
5 – HT: 5 – hydroxytryptamine
PYY: peptide tyrosine tyrosine
SCFA: short-chain fatty acids
TMAO: trimethylamine N-oxide
BBB: blood-brain barrier

## References

[1] Roszkowski A (2011). Technologie produkcji zwierzęcej a emisje gazów cieplarnianych. Problemy Inżynierii Rolniczej.

[2] Khafipour E, Li S, Tun HM, Derakhshani H, Moossavi S, Plaizier JC (2016). Effects of grain feeding on microbiota in the digestive tract of cattle. Anim Front.

[3] O’Hara E, Neves AL, Song Y, Guan LL (2020). The Role of the Gut Microbiome in Cattle Production and Health: Driver or Passenger?. Ann Rev Anim Biosci.

[4] Jami E, White BA, Mizrahi I (2014). Potential role of the bovine rumen microbiome in modulating milk composition and feed efficiency. PLoS One.

[5] Young EI, Contain Multitudes. The Microbes Within Us and a Grander View of Life, Random House, 2016. 235–246 [PL version].

[6] de Nardi R, Marchesini G, Li S, Khafipour E, Plaizier KJ, Gianesella M, Ricci R, Andrighetto I, Segato S (2016). Metagenomic analysis of rumen microbial population in dairy heifers fed a high grain diet supplemented with dicarboxylic acids or polyphenols. BMC Vet Res.

[7] Matthews C, Crispie F, Lewis E, Reid M, O’Toole PW, Cotter PD (2019). The rumen microbiome: a crucial consideration when optimising milk and meat production and nitrogen utilisation efficiency. Gut Microbes.

[8] Rey M, Enjalbert F, Combes S, Cauquil L, Bouchez O, Monteils V (2013). Establishment of ruminal bacterial community in dairy calves from birth to weaning is sequential. J Appl Microbiol.

[9] Wang L, Zhang K, Zhang C, Feng Y, Zhang X, Wang X, Wu G (2019). Dynamics and stabilization of the rumen microbiome in yearling Tibetan sheep. Sci Rep.

[10] Cholewińska P, Czyż K, Nowakowski P, Wyrostek A. The microbiome of the digestive system of ruminants – a review. Ani Health Res Rev. 2020, 1–12. 10.1017/S1466252319000069 31918781

[11] Armstrong D (1994). Heat stress interaction with shade and cooling. J Dairy Sci.

[12] Kadzere CT, Murphy MR, Silanikove N, Maltz E (2002). Heat stress in lactating dairy cows: a review. Livestock Prod Sci.

[13] Salcedo J, Frese SA, Mills DA, Barile D (2016). Characterization of porcine milk oligosaccharides during early lactation and their relation to the fecal microbiome. J Dairy Sci.

[14] Henderson G, Cox F, Ganesh S, Jonker A, Young W, Abecia L, Attwood G (2015). T. Rumen microbial community composition varies with diet and host, but a core microbiome is found across a wide geographical range. Sci Rep.

[15] Allison MJ, Mayberry WR, Mcsweeney CS, Stahl DA (1992). Synergistes jonesii, gen. nov., sp. nov.: a rumen bacterium that degrades toxic pyridinediols. Syst Appl Microbiol.

[16] Clark B, Stewart GB, Panzone LA, Kyriazakis I, Frewer LJ (2017). Citizens, consumers and farm animal welfare: A meta-analysis of willingness-to-pay studies. Food Policy.

[17] Ritter C, Beaver A, von Keyserlingk MA (2019). The complex relationship between welfare and reproduction in cattle. Reprod Domest Anim.

[18] Fonty G, Gouet P, Jouany JP, Senaud J (1987). Establishment of the microflora and anaerobic fungi in the rumen of lambs. Microbiology.

[19] Gabryszuk M, Sakowski T, Metera E, Kuczynska B, Rembialkowska E (2013). Effect of Feeding on Content of Bioactive Substances in Milk from Cows Raised in Organic Farms. Zywnosc-Nauka Technologia Jakosc.

[20] Hernandez-Sanabria E, Goonewardene LA, Li M, Mujibi DF, Stothard P, Moore SS, Leon-Quintero MC (2010). Correlation of particular bacterial PCR-denaturing gradient gel electrophoresis patterns with bovine ruminal fermentation parameters and feed efficiency traits. Appl Environ Microbiol.

[21] de Nadra MM. Nitrogen metabolism in lactic acid bacteria from fruits: a review. Communicating Current Research and Educational Topics and Trends in Applied Microbiology 2007, Badajoz, Formatex, 500–510.

[22] Williams AG, Coleman GS (1997). The rumen protozoa. The rumen microbial ecosystem.

[23] Clauss M, Müller K, Fickel J, Streich WJ, Hatt JM, Südekum KH (2011). Macroecology of the host determines microecology of endobionts: protozoal faunas vary with wild ruminant feeding type and body mass. J Zool.

[24] Jouany JP (1996). Effect of rumen protozoa on nitrogen utilization by ruminants. J Nutr.

[25] Benchaar C, McAllister TA, Chouinard PY, Digestion (2018). ruminal fermentation, ciliate protozoal populations, and milk production from dairy cows fed cinnamaldehyde, quebracho condensed tannin, or Yucca schidigera saponin extracts. J Dairy Sci.

[26] Vďačný P, Orsi W, Bourland WA, Shimano S, Epstein SS, Foissner W (2011). Morphological and molecular phylogeny of dileptid and tracheliid ciliates: Resolution at the base of the class Litostomatea (Ciliophora, Rhynchostomatia). Eur J Protistol.

[27] Gao F, Warren A, Zhang Q, Gong J, Miao M, Sun P, Xu D, Huang J, Yi Z, Song W (2016). The all-data-based evolutionary hypothesis of ciliated protists with a revised classification of the phylum Ciliophora (Eukaryota, Alveolata). Sci Rep.

[28] McCann JC, Wickersham TA, Loor JJ. High-throughput methods redefine the rumen microbiome and its relationship with nutrition and metabolism. Bioinformatics and biology insights. 2014, 8, BBI. S15389. 10.4137/BBI.S15389 PMC4055558 24940050

[29] Khafipour E, Li S, Plaizier JC, Krause DO (2009). Rumen microbiome composition determined using two nutritional models of subacute ruminal acidosis. Appl Environ Microbiol.

[30] Hempel S, Menz C, Pinto S, Galán E, Janke D, Estellés F, Müschner-Siemens T, Wang X, Heinicke J, Zhang G, Amon B (2009). Heat stress risk in European dairy cattle husbandry under different climate change scenarios–uncertainties and potential impacts. Earth Sys Dyn.

[31] Kassow A, Rahmann G, Blank B, Paulsen HM, Aulrich K. Studies on greenhouse gas emissions in organic and conventional dairy farms. In Ressortforschung für den Ökologischen Landbau 2009, 65–76. Johann Heinrich von Thünen-Institut- Bundesforschungsinstitut für Ländliche 2010.

[32] Moss AR, Jouany JP, Newbold J. Methane production by ruminants: its contribution to global warming. In Annales de zootechnie. 2000, 231–253. EDP Sciences.

[33] Janssen PH, Kirs M (2008). Structure of the archaeal community of the rumen. Appl Environ Microbiol.

[34] McAllister TA, Cheng KJ, Okine EK, Mathison GW (1996). Dietary, environmental and microbiological aspects of methane production in ruminants. Canadian Journal of Animal Science.

[35] De Menezes AB, Lewis E, O’Donovan M, O’Neill BF, Clipson N, Doyle EM (2011). Microbiome analysis of dairy cows fed pasture or total mixed ration diets. FEMS Microbiol Ecol.

[36] Kirchgessner M. Nutritional factors for the quantification of methane production. Ruminant Physiology: Digestion, Methabolism, Growth and Reproduction. 1995, 317–331.

[37] Michelland RJ, Monteils V, Combes S, Cauquil L, Gidenne T, Fortun-Lamothe L (2010). Comparison of the archaeal community in the fermentative compartment and faeces of the cow and the rabbit. Anaerobe.

[38] Zhou M, Chung YH, Beauchemin KA, Holtshausen L, Oba M, McAllister TA, Guan LL (2011). Relationship between rumen methanogens and methane production in dairy cows fed diets supplemented with a feed enzyme additive. J Appl Microbiol.

[39] Kong Y, Xia Y, Seviour R, Forster R, McAllister TA (2013). Biodiversity and composition of methanogenic populations in the rumen of cows fed alfalfa hay or triticale straw. FEMS Microbiol Ecol.

[40] Abecia L, Martín-García AI, Martínez G, Newbold CJ, Yáñez-Ruiz DR (2013). Nutritional intervention in early life to manipulate rumen microbial colonization and methane output by kid goats postweaning. J Anim Sci.

[41] Wolin MJ. The rumen fermentation: a model for microbial interactions in anaerobic ecosystems. In Advances in microbial ecology. 1979, 49–77. Springer.

[42] O’Callaghan TF, Ross RP, Stanton C, Clarke G (2016). The gut microbiome as a virtual endocrine organ with implications for farm and domestic animal endocrinology. Domest Anim Endocrinol.

[43] Yáñez-Ruiz DR, Abecia L, Newbold CJ (2015). Manipulating rumen microbiome and fermentation through interventions during early life: a review. Front Microbiol.

[44] Mao SY, Huo WJ, Zhu WY (2016). Microbiome–metabolome analysis reveals unhealthy alterations in the composition and metabolism of ruminal microbiota with increasing dietary grain in a goat model. Environ Microbiol.

[45] Bhattarai Y, Williams BB, Battaglioli EJ, Whitaker WR, Till L, Grover M, Kaunitz JD (2018). Gut microbiota-produced tryptamine activates an epithelial G-protein-coupled receptor to increase colonic secretion. Cell Host Microbe.

[46] Fernando SC, Purvis HT, Najar FZ, Sukharnikov LO, Krehbiel CR, Nagaraja TG, DeSilva U (2010). Rumen microbial population dynamics during adaptation to a high-grain diet. Appl Environ Microbiol.

[47] Li S, Khafipour E, Krause DO, Rodriguez-Lecompte JC, Plaizier JC (2010). Free endotoxins in the feces of lactating dairy cows. Can J Anim Sci.

[48] Madejska A, Michalski M, Osek J. Aminy biogenne w serach podpuszczkowych dojrzewających jako zagrożenie zdrowia konsumentów. Medycyna Weterynaryjna. 2017, 214–219.

[49] Li M, Zhou M, Adamowicz E, Basarab JA (2012). Characterization of bovine ruminal epithelial bacterial communities using 16S rRNA sequencing, PCR-DGGE, and qRT-PCR analysis. Vet Microbiol.

[50] Tamate H, McGilliard A, Jacobson N, Getty R (1962). Effect of various dietaries on the anatomical development of the stomach in the calf. J Dairy Sci.

[51] Addis MF, Tanca A, Uzzau S, Oikonomou G, Bicalho RC, Moroni P (2016). The bovine milk microbiota: insights and perspectives from-omics studies. Mol BioSyst.

[52] Meale SJ, Li S, Azevedo P, Derakhshani H, Plaizier JC, Khafipour E, Steele MA (2016). Development of ruminal and fecal microbiomes are affected by weaning but not weaning strategy in dairy calves. Front Microbiol.

[53] Gauly M, Bollwein H, Breves G, Brügemann K, Dänicke S, Daş G, Demeler J, Hansen H, Isselstein J, König S, Lohölter M (2013). Future consequences and challenges for dairy cow production systems arising from climate change in Central Europe–a review. Animal.

[54] Hansen PJ, Drost M, Rivera RM, Paula-Lopes FF, Al-Katanani YM, Krininger IIICE (2001). Chase C.C. Adverse impact of heat stress on embryo production causes and strategies for mitigation. Theriogenology.

[55] Baumgard LH, Seibert JT, Kvidera SK, Keating AF, Ross JW, Rhoads RP (2016). Production, biological, and genetic responses to heat stress in ruminants and pigs. J Anim Sci.

[56] Uyeno Y, Sekiguchi Y, Tajima K, Takenaka A, Kurihara M, Kamagata Y (2010). An rRNA- based analysis for evaluating the effect of heat stress on the rumen microbial composition of Holstein heifers. Anaerobe.

[57] Contreras-Jodar A, Nayan NH, Hamzaoui S, Caja G, Salama AA (2019). Heat stress modifies the lactational performances and the urinary metabolomic profile related to gastrointestinal microbiota of dairy goats. PLOS ONE.

[58] Gantner V, Bobić T, Gregić M, Gantner R, Kuterovac K, Potočnik K. The differences in heat stress resistance due to dairy cattle breed. Mljekarstvo: časopis za unaprjeđenje proizvodnje i prerade mlijeka 2017, 19; 67(2):112–22.

[59] Gart EV, Lawhon SD, Suchodolski JS, Randel RD, Welsh TH (2019). The relationship of weaning stress, sex and temperament on fecal microbiota and metabolites in Brahman calves. J Anim Sci.

[60] Jami E, Israel A, Kotser A (2013). Mizrahi I.Exploring the bovine rumen bacterial community from birth to adulthood. ISME J.

[61] Tajima K, Nonaka I, Higuchi K, Takusari N, Kurihara M, Takenaka A, Mitsumori M, Kajikawa H (2007). Aminov R. I. Influence of high temperature and humidity on rumen bacterial diversity in Holstein heifers. Anaerobe.

[62] Chen S, Wang J, Peng D, Li G, Chen J, Gu X (2018). Exposure to heat-stress environment affects the physiology, circulation levels of cytokines, and microbiome in dairy cows. Sci Rep.

[63] Kokocinska A, Kaleta T. Znaczenie etologii w naukach o dobrostanie zwierząt. Roczniki Naukowe Polskiego Towarzystwa Zootechnicznego 2016, 12(1).

[64] Deng L, He C, Zhou Y, Xu L, Xiong H (2017). Ground transport stress affects bacteria in the rumen of beef cattle: A real-time PCR analysis. Anim Sci J.

[65] Salhi S, Bouzebda-Afri F, Bouzebda Z, Djaout A, Ouenes H. Study of the biochemical parameters of pre-slaughter stress response in bovine species in algeria. Advances in Animal and Veterinary Sciences 2020, 8.

[66] Foster JA, Rinaman L, Cryan JF (2017). Stress & the gut–brain axis: Regulation by the microbiome. Neurobiology Stress.

[67] Muller PA, Schneeberger M, Matheis F, Wang P, Kerner Z, Ilanges A, Pellegrino K, Del Mármol J, Castro TB, Furuichi M, Perkins M. Microbiota modulate sympathetic neurons via a gut–brain circuit. Nature. 2020, 1–6. 10.1038/s41586-020-2474-7 PMC7367767 32641826

[68] Bailey MT Psychological stress, immunity, and the effects on indigenous microflora. In Microbial endocrinology: Interkingdom signaling in infectious disease and health (225–246). Springer. 2016, Cham. 10.1007/978-3-319-20215-0_11 26589222

[69] Freestone P, Lyte M (2010). Stress and microbial endocrinology: prospects for ruminant nutrition. Animal.

[70] Wang SZ, Yu YJ, Adeli K (2020). Role of Gut Microbiota in Neuroendocrine Regulation of Carbohydrate and Lipid Metabolism via the Microbiota-Gut-Brain-Liver Axis. Microorganisms.

[71] Wang P, Daotong L, Weixin K, Dong L, Xiaosong H, Fang C (2020). Resveratrol-induced gut microbiota reduces obesity in high-fat diet-fed mice. Int J Obes.

[72] Mao JH, Kim YM, Zho YX, Hu D, Zhong C, Chang H, Brislawn C, Langley S, Wang Y, Peisl BL, Celniker SE (2020). Genetic and metabolic links between the murine microbiome and memory. Microbiome.

[73] Obata O, Salar-Garcia MJ, Greenman J, Kurt H, Chandran K, Ieropoulos I (2020). Development of efficient electroactive biofilm in urine-fed microbial fuel cell cascades for bioelectricity generation. J Environ Manage.

[74] Fung T, Olson C, Hsiao E (2017). Interactions between the microbiota, immune and nervous systems in health and disease. Nat Neurosci.

[75] Liu WH, Chuang HL, Huang YT, Wu CC, Chou GT, Wang S, Tsai YC (2016). Alteration of behavior and monoamine levels attributable to Lactobacillus plantarum PS128 in germ-free mice. Behav Brain Res.

[76] Desbonnet L, Clarke G, Traplin A, O’Sullivan O, Crispie F, Moloney RD, Cotter PD, Dinan TG, Cryan JF (2015). Gut microbiota depletion from early adolescence in mice: implications for brain and behaviour. Brain Behav Immun.

[77] Hyland NP, Cryan JF (2016). Microbe-host interactions: Influence of the gut microbiota on the enteric nervous system. Dev Biol.

[78] Hornby PJ, Moore BA (2011). The therapeutic potential of targeting the glucagon-like peptide-2 receptor in gastrointestinal disease. Expert Opin Ther Targets.

[79] Reiche EM, Nunes SO, Morimoto HK (2004). Stress, depression, the immunesystem, and cancer. Lancet Oncol.

[80] Castellazzi A, Tagliacarne SC, Soldi S, Perna S, Ziviani L, Milleri S, Valsecchi C (2018). Stress and immune function: there is a role for the gut microbiota?. J Clin Gastroenterol.

[81] Alldritt I, Whitham-Agut B, Sipin M, Studholme J, Trentacoste A, Tripp JA, Cappai MG, Ditchfield P, Devièse T, Hedges RE (2019). McCullagh J.S. Metabolomics reveals diet-derived plant polyphenols accumulate in physiological bone. Sci Rep.

